# Allergen-specific IgE sensitization spectrum in 4,166 children with allergic diseases at a county-level hospital in eastern China: a retrospective cross-sectional study

**DOI:** 10.3389/falgy.2026.1893168

**Published:** 2026-09-07

**Authors:** Yan Teng, Shouling Ding, Baoqin Zhang

**Affiliations:** Department of Pediatrics, The First People’s Hospital of Taicang City, Suzhou, China

**Keywords:** allergens, children, co-sensitization, county-level hospital, house dust mite, specific IgE

## Abstract

**Objective:**

This study aimed to characterize the serum specific IgE sensitization spectrum in children with allergic diseases at a county-level tertiary hospital and to evaluate age-, sex-, and co-sensitization patterns.

**Methods:**

This single-center retrospective cross-sectional study included 4,166 children aged <18 years with allergic diseases who underwent serum specific IgE (sIgE) testing at the First People's Hospital of Taicang City between 2019 and 2024. Twenty allergens (10 inhalant and 10 ingested) were measured by enzyme immunoassay, with sensitization defined as sIgE ≥0.35 kU/L. Sensitization patterns by sex and age were assessed using *χ*^2^/Fisher's exact tests and multivariable logistic regression, and co-sensitization was evaluated using the *φ* coefficient.

**Results:**

The overall sensitization rate was 75.85%, with higher positivity in boys than girls (77.46% vs. 73.49%, *P* = 0.004); polysensitization was present in 57.35% of children (33.20%, 65.80%, and 56.99% in the <3, 3 to <12, and 12 to <18 years groups, respectively). The most common allergens were egg, *Dermatophagoides farinae*, house dust, *Dermatophagoides pteronyssinus*, and cow's milk. Sensitization showed a clear age-related shift from food allergens in children <3 years to inhalant allergens in adolescents. Male sex independently increased the risk of overall sensitization (aOR 1.26, 95% CI 1.09–1.46). Sensitization to mites and cat epithelium increased with age, whereas sensitization to egg and cow's milk declined. Co-sensitization analysis identified a strong mite cluster (*D. farinae* and *D. pteronyssinus*, *φ* = 0.888), a mite-related house-dust cluster, and a moderate early-food cluster involving egg, cow's milk, and wheat flour.

**Conclusion:**

In this county-level cohort from the northern Yangtze River Delta, pediatric allergen sensitization was dominated by house dust mites and early food allergens, with sex- and age-related differences and distinct co-sensitization patterns. These findings may inform allergen screening and pediatric allergy management in similar regional settings.

## 1 Introduction

Allergic diseases have become one of the most prevalent chronic conditions in children worldwide. In China, the prevalence of asthma in urban children rose from 0.91% in 1990 to 3.02% in 2010, and the self-reported prevalence of allergic rhinitis increased from 11.1% to 17.6% between 2005 and 2011 (1, 2). House dust mites (HDMs) and early food allergens such as egg and cow's milk have long occupied the core of the sensitization spectrum in Chinese children, but the specific composition is strongly shaped by geography and climate. Mite sensitization predominates along the humid eastern and southern coasts, whereas pollen sensitization to *Artemisia* and ragweed predominates in the arid and semi-arid northwest (3–5). The pediatric allergen spectrum is not static; it evolves with age along the classical “food-to-respiratory” trajectory known as the atopic march (6, 7).

Large-scale Chinese studies of pediatric sensitization have predominantly been conducted at tertiary referral centers in major cities, whereas data from county-level hospitals remain limited. Extrapolating such findings may not adequately inform local decisions on allergen screening, dietary avoidance, referral, and allergen immunotherapy (AIT). Taicang City, located in the humid Yangtze River Delta, has climatic conditions favorable for mites and molds and an increasingly urbanized lifestyle that may shape allergen exposure. We analyzed 4,166 children with allergic diseases attending the First People' s Hospital of Taicang City from 2019 to 2024 to characterize sensitization to 20 common allergens and associated age-, sex-, and co-sensitization patterns, with the aim of informing pediatric allergy assessment and management in similar regional settings.

## 2 Materials and methods

### 2.1 Study design and participants

This was a single-center retrospective cross-sectional study. Data were retrieved from the laboratory information system (LIS) and the electronic medical record system of the First People's Hospital of Taicang City. Eligible participants were children aged 0 to <18 years who attended the pediatric outpatient or inpatient department between 1 January 2019 and 31 December 2024 and who underwent serum IgE testing, including at least one panel of sIgE testing, during the same encounter. Data were extracted by one reviewer using a standardized data-collection form covering demographic characteristics, primary encounter diagnosis, sIgE results, testing panel, and eligibility variables, and were independently checked by a second reviewer against the source LIS and electronic medical record fields. Any discrepancies or uncertainties identified during the checking process were cross-reviewed by a third reviewer through re-examination of the source records, and the final dataset was established after consensus was reached.

**Inclusion criteria:** (1) aged 0 to <18 years at first presentation; (2) a clinical diagnosis or working diagnosis of an allergic disease, including allergic rhinitis, bronchial asthma, atopic dermatitis, eczema, urticaria, acute wheezy bronchitis, allergic conjunctivitis, or angioedema; and (3) availability of at least one eligible sIgE panel result during the study period. Clinical diagnoses were made according to the diagnostic criteria outlined in *Practical Pediatrics* (8); because the retrieved variable was the primary encounter diagnosis, diagnosis-stratified sensitization rates are reported in Section 3.1.

**Exclusion criteria:** (1) receipt of systemic corticosteroids, omalizumab, or other biological agents within 6 months before enrollment; (2) coexisting primary immunodeficiency, malignancy, active autoimmune disease, or chronic hepatic or renal insufficiency; and (3) missing values for key variables (sex, age at testing, or the sIgE result analyzed) or evident logical inconsistencies; no imputation was used. For children with repeated sIgE measurements during the study period, only the first eligible result for each panel was retained.

The study involving humans was approved by the Ethics Committee of the First People's Hospital of Taicang City (approval number 2025-SR-011). Because this was a retrospective analysis of de-identified records obtained during routine care, the Ethics Committee approved the study and waived the requirement for retrospective written informed consent; all records were de-identified before analysis.

### 2.2 Laboratory assays

Serum sIgE was quantified by enzyme immunoassay (EIA) on an HB-500E automated immunoassay analyzer (Coredeo Medical, China) using the manufacturer's matched allergen sIgE detection kits (Hob Biotech, China). Twenty allergens were assessed, including 10 inhalant allergens [*Dermatophagoides pteronyssinus*, *Dermatophagoides farinae*, house dust, mugwort, willow, common ragweed, German cockroach (*Blattella germanica*), cat epithelium, dog epithelium, and *Alternaria alternata*] and 10 ingested allergens (egg, cow's milk, wheat flour, peanut, shrimp, crab, soybean, codfish, beef, and mutton). All measurements were performed strictly in accordance with the manufacturer's instructions, and inter-batch variation of the reagents was monitored by routine laboratory quality control. The “house dust” reagent is a whole-dust extract that contains mite-derived proteins and is therefore not biologically independent of the two *Dermatophagoides* species. Serum tIgE was measured on a Roche cobas e601 electrochemiluminescence immunoassay platform (Roche Diagnostics), and elevation was defined relative to the age-specific upper limit of normal (ULN) provided by the manufacturer. sIgE concentrations were stratified according to the seven-level grading scheme of the Phadia ImmunoCAP system (Thermo Fisher Scientific, Uppsala, Sweden) into seven levels (9): class 0 (<0.35 kU/L, negative); class 1 (0.35 to 0.69 kU/L); class 2 (0.70 to 3.49 kU/L); class 3 (3.50 to 17.49 kU/L); class 4 (17.50 to 49.99 kU/L); class 5 (50.00 to 99.99 kU/L); and class 6 (≥100.00 kU/L). Single-allergen positivity in this study was defined as sIgE ≥0.35 kU/L (i.e., class 1 or above).

### 2.3 Variable definitions

1. **Age stratification:** based on the clinical staging of the natural course of pediatric allergic disease, participants were grouped into <3 years, 3 to <12 years, and 12 to <18 years. These categories were selected *a priori* with reference to the natural history and clinical staging of pediatric allergic diseases and previous Chinese pediatric allergen studies (10).
2. **Total sensitization:** sIgE ≥ class 1 to any one of the 20 allergens tested.
3. **Polysensitization:** sIgE ≥ class 1 to two or more allergens.
4. **Number of sensitizations:** grouped into 0, 1, 2 to 4, 5 to 7, and ≥8 to describe the distribution of sensitization burden.
5. **Co-sensitization:** simultaneous positivity to two allergens, with the pairwise correlation strength quantified by the *φ* coefficient.

### 2.4 Statistical analysis

All analyses were performed in Python 3.12 using pandas 2.2, SciPy 1.13, and statsmodels 0.14, with key estimates re-run independently in SPSS 27.0 (IBM, Armonk, NY, USA) with identical results; this step verifies software implementation and is not statistical cross-validation. Normality was assessed using the Shapiro–Wilk test. Normally distributed variables were expressed as mean ± SD and compared using Student's t-test or one-way ANOVA; non-normally distributed variables were expressed as median (IQR) and compared using the Mann–Whitney U or Kruskal–Wallis H test. Categorical variables were expressed as *n* (%) and compared using Pearson's *χ*^2^ or Fisher's exact test, as appropriate. Total sensitization was calculated for the entire cohort, whereas allergen-specific and sex-/age-stratified rates were based on children tested for the corresponding panel; untested children were not considered negative. As the 20 allergen comparisons were prespecified descriptive analyses, primary results are presented without adjustment, with Benjamini–Hochberg FDR-adjusted *q* values reported for sex and age comparisons.

Binary logistic regression was used to identify independent determinants of overall or allergen-specific sensitization, with sex (female reference) and age group (<3 years reference) as independent variables; aORs and 95% CIs are reported. Because the models contained only sex and age group (six covariate patterns), the Hosmer–Lemeshow statistic was computed over these patterns, and VIF, a sex-by-age likelihood-ratio test, and *post-hoc* power for the adolescent subgroup are reported. No *a priori* sample-size calculation was performed because all eligible records were analyzed. Season and year were not modeled because sIgE reflects cumulative sensitization rather than an acute seasonal response, and the study describes the pooled six-year profile; a sensitivity analysis added diagnostic category to the total-sensitization model.

Co-sensitization between allergen pairs was assessed using 2 × 2 contingency tables. The strength of association was quantified using the *φ* coefficient, and statistical significance was evaluated using Pearson's *χ*^2^ test or Fisher's exact test when expected cell counts were small. The *φ* coefficient, equivalent to the Pearson correlation coefficient for binary variables in a 2 × 2 contingency table, ranges from −1 to +1 and reflects the strength of association between the sensitization statuses of two allergens. Following Cohen's conventional thresholds, |*φ*| ≥ 0.20 was interpreted as a moderate association and |*φ*| ≥ 0.50 as a strong association. All statistical tests were two-sided, and *P* < 0.05 was considered statistically significant.

## 3 Results

### 3.1 Demographic characteristics

A total of 4,234 children were assessed for eligibility. Of these, 62 were excluded because of systemic corticosteroid or biologic therapy within the preceding 6 months, including 2 who had received omalizumab, and 6 were excluded because of the prespecified comorbidities. The final study cohort therefore comprised 4,166 children with allergic diseases. Among these, 119 children (2.9%) underwent repeated sIgE panel testing during the study period. After retaining only the first eligible result for each panel, 216 repeat panels (109 inhalant and 107 ingested panels) were excluded from the analysis. The cohort included 2,476 boys (59.43%) and 1,690 girls (40.57%), with a male-to-female ratio of approximately 1.47:1. The median age was 4.0 years (IQR 3.0 to 7.0 years). By age group, 1,030 children (24.72%) were <3 years, 2,950 (70.81%) were 3 to <12 years, and 186 (4.47%) were 12 to <18 years. Inhalant sIgE testing was completed in 3,758 children and ingested sIgE testing in 3,809 children; both panels were performed in 3,401 children (81.6%). The leading clinical diagnoses, in descending order, were acute bronchitis, urticaria, eczema, bronchial asthma, and acute wheezy bronchitis. Diagnosis-stratified total sensitization was 87.1% for allergic rhinitis/rhinitis (*n* = 317), 85.0% for bronchial asthma (*n* = 280), 78.6% for non-wheezy acute respiratory infection including acute bronchitis (*n* = 1,129), and 70.6% for eczema, dermatitis, or urticaria (*n* = 1,269; *χ*^2^ = 58.51, *P* < 0.001); the acute respiratory infection group was not less sensitized than the rest (78.6% vs. 75.9%, *P* = 0.084). Among children with respiratory diagnoses, mite sensitization and overall inhalant sensitization were observed in 46.0% and 64.5%, respectively, whereas the corresponding rates among children with eczema, dermatitis, or urticaria were 33.3% and 58.2%. Sensitization to early food allergens (egg and cow's milk) was also common in both diagnostic groups (60.5% and 57.5%, respectively).

### 3.2 Overall positivity of tIgE and sIgE

tIgE was measured in 3,322 children, with median values of 87.3, 138.3, and 191.5 IU/mL across the three age groups (*P* < 0.001). The proportion of children with elevated tIgE was significantly higher in boys (69.03%) than in girls (56.51%; *P* < 0.001). The overall sIgE positivity was 75.85% (3,160/4,166), and was significantly higher in boys (77.46%) than in girls (73.49%; *χ*^2^ = 8.438, *P* = 0.004). Boys had significantly higher positivity than girls for nine allergens, namely *D. pteronyssinus*, *D. farinae*, house dust, *A. alternata*, cat epithelium, dog epithelium, willow, shrimp, and crab. No significant sex difference was observed for major ingested allergens such as egg and cow's milk (Table 1). After BH-FDR correction, six differences remained significant (*q* ≤ 0.049); cat epithelium, dog epithelium, and shrimp did not (*q* = 0.087, 0.098, and 0.100) and are treated as hypothesis-generating.

**Table 1:** Comparison of sIgE positivity rates between male and female children across allergens.

Allergen	Male [n (%)]	Female [n (%)]	*P*	BH-FDR *q*
Total positive	1,918 (77.46)	1,242 (73.49)	0.004	—
**Inhalant allergens**				
*Dermatophagoides farinae*	933 (41.65)	547 (36.03)	<0.001	0.004
*Dermatophagoides pteronyssinus*	899 (40.13)	510 (33.60)	<0.001	0.001
House dust	898 (40.09)	535 (35.24)	0.003	0.011
*Alternaria alternata*	489 (21.83)	250 (16.47)	<0.001	0.001
Cat epithelium	184 (8.21)	96 (6.32)	0.030	0.087
Dog epithelium	106 (4.73)	51 (3.36)	0.039	0.098
Willow	23 (1.03)	5 (0.33)	0.015	0.049
Common ragweed	29 (1.29)	11 (0.72)	0.095	0.184
Mugwort	16 (0.71)	5 (0.33)	0.120	0.201
German cockroach (*Blattella germanica*)	4 (0.18)	3 (0.20)	1.000	1.000
**Ingested allergens**				
Egg	1,063 (47.08)	722 (46.55)	0.749	0.881
Cow's milk	847 (37.51)	577 (37.20)	0.846	0.940
Wheat flour	298 (13.20)	177 (11.41)	0.101	0.184
Crab	123 (5.45)	51 (3.29)	0.002	0.009
Shrimp	69 (3.06)	31 (2.00)	0.045	0.100
Codfish	59 (2.61)	40 (2.58)	0.948	0.998
Peanut	47 (2.08)	24 (1.55)	0.231	0.308
Soybean	40 (1.77)	21 (1.35)	0.313	0.392
Beef	27 (1.20)	11 (0.71)	0.138	0.212
Mutton	4 (0.18)	0 (0.00)	0.151	0.215

Total positivity denominators: male *n* = 2,476, female *n* = 1,690. Allergen-specific percentages use sex-specific tested n: inhalant male/female 2,240/1,518; ingested 2,258/1,551. Untested children were excluded. The final column gives Benjamini–Hochberg adjusted q values for the 20 allergen comparisons; total positivity was not included in this family.

### 3.3 Distribution of sIgE positivity classes by allergen

**Figure 1:**
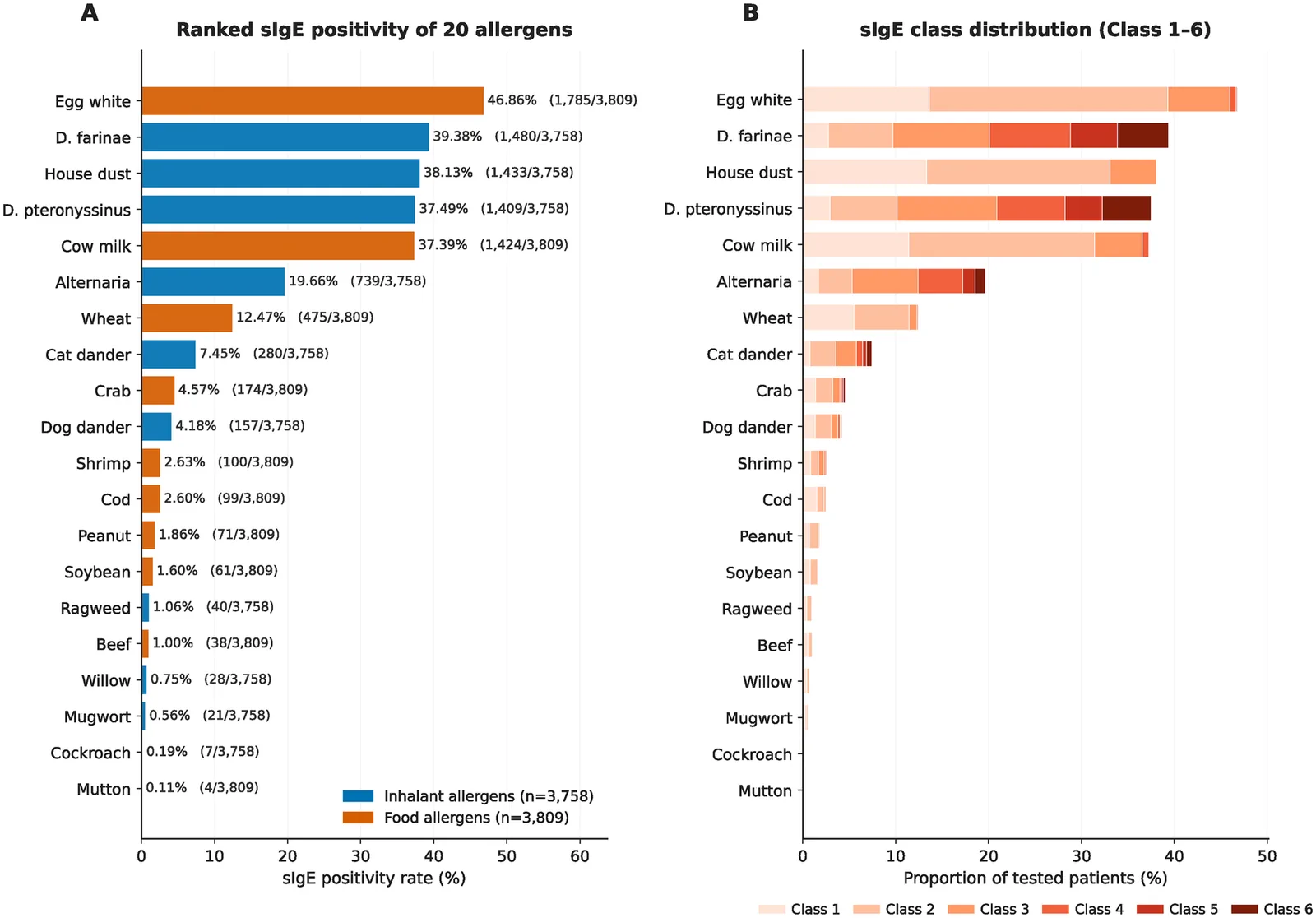
Sensitization spectrum and sIgE class stratification across 20 allergens. **(A)** Horizontal bar chart showing the 20 allergens ranked by sIgE positivity (blue, inhalant allergens, *n* = 3,758; orange, ingested allergens, *n* = 3,809). **(B)** Stacked distribution of sIgE classes (1 to 6) for the same allergen order.

The five most frequently positive allergens were egg (46.86%), *D. farinae* (39.38%), house dust (38.13%), *D. pteronyssinus* (37.49%), and cow's milk (37.39%). With respect to grade distribution, high-class sensitization (classes 4 to 6) was disproportionately frequent for *D. farinae*, *D. pteronyssinus*, *A. alternata*, cat epithelium, and crab, suggesting that these allergens may elicit clinically more potent sensitization. In contrast, sensitization to egg, cow's milk, house dust, and wheat flour was dominated by low classes (1 to 2), indicating that most of the children sensitized to these allergens were in a relatively mild or early stage of sensitization (Table 2**,** Figure 1).

**Table 2:** Distribution of sIgE positivity classes for each allergen [*n*].

Allergen	Total tested (*n*)	Positive *n* (%)	Class 1	Class 2	Class 3	Class 4	Class 5	Class 6
**Inhalant allergens**								
*Dermatophagoides farinae*	3,758	1,480 (39.38)	104	260	390	328	191	207
*Dermatophagoides pteronyssinus*	3,758	1,409 (37.49)	110	271	403	276	152	197
House dust	3,758	1,433 (38.13)	500	742	189	2	0	0
*Alternaria alternata*	3,758	739 (19.66)	63	136	267	180	50	43
Cat epithelium	3,758	280 (7.45)	29	105	83	24	16	23
Dog epithelium	3,758	157 (4.18)	50	65	27	8	2	5
Common ragweed	3,758	40 (1.06)	16	20	4	0	0	0
Willow	3,758	28 (0.75)	16	11	1	0	0	0
Mugwort	3,758	21 (0.56)	10	6	4	0	0	1
German cockroach (*Blattella germanica*)	3,758	7 (0.19)	6	1	0	0	0	0
**Ingested allergens**								
Egg	3,809	1,785 (46.86)	519	977	255	25	5	4
Cow's milk	3,809	1,424 (37.39)	434	762	196	26	3	3
Wheat flour	3,809	475 (12.47)	210	226	31	5	3	0
Crab	3,809	174 (4.57)	53	69	31	6	6	9
Shrimp	3,809	100 (2.63)	32	32	23	7	2	4
Codfish	3,809	99 (2.60)	57	31	6	2	3	0
Peanut	3,809	71 (1.86)	27	37	5	1	1	0
Soybean	3,809	61 (1.60)	31	29	1	0	0	0
Beef	3,809	38 (1.00)	20	18	0	0	0	0
Mutton	3,809	4 (0.11)	2	2	0	0	0	0

Class 0 (<0.35 kU/L) is not shown; percentages use children tested for the corresponding panel. “House dust” is a composite extract containing mite-derived proteins (Sections 2.2, 4.1).

### 3.4 Age-stratified sensitization and polysensitization

The overall sensitization rates in the three age groups were 58.74%, 82.20%, and 69.89% (*P* < 0.001), forming a school-age peak with adolescent decline. In the <3 years group, cow's milk (46.75%) and egg (43.18%) and other ingested allergens predominated. In the 3 to <12 years group, the spectrum became a balanced mixture of inhalant and ingested allergens, with the leading positivities being egg (49.48%), *D. farinae* (45.32%), *D. pteronyssinus* (42.86%), house dust (40.03%), and cow's milk (36.50%). In the 12 to <18 years group, the spectrum shifted toward an inhalant-dominant profile, with the top five being *D. pteronyssinus* (49.72%), *D. farinae* (47.49%), house dust (40.22%), cat epithelium (17.32%), and *A. alternata* (16.76%; Table 3). The distribution of the number of sensitizations was as follows: 0 in 1,006 children (24.15%), 1 in 771 (18.51%), 2 to 4 in 1,695 (40.69%), 5 to 7 in 619 (14.86%), and ≥8 in 75 (1.80%). Polysensitization (≥2 allergens) was observed in 57.35% of the cohort, with the proportion in the 3 to <12 years group being significantly higher than in the other two groups (*χ*^2^ = 379.432, *P* < 0.001; Table 4). In the 3,401 children with both panels, total sensitization was 81.2% overall (75.7%, 82.7%, and 69.9% by age group) and polysensitization was 64.4%. All age-group differences that were significant before adjustment remained significant after BH-FDR correction (*q* ≤ 0.048).

**Table 3:** Comparison of sIgE positivity rates across age groups [*n* (%)].

Allergen	<3 years (*n* = 1,030)	3 to <12 years (*n* = 2,950)	12 to <18 years (*n* = 186)	*P*
Total positive	605 (58.74)	2,425 (82.20)	130 (69.89)	<0.001
**Inhalant allergens**				
*Dermatophagoides farinae*	84 (12.24)	1,311 (45.32)	85 (47.49)	<0.001
*Dermatophagoides pteronyssinus*	80 (11.66)	1,240 (42.86)	89 (49.72)	<0.001
House dust	203 (29.59)	1,158 (40.03)	72 (40.22)	<0.001
*Alternaria alternata*	32 (4.66)	677 (23.40)	30 (16.76)	<0.001
Cat epithelium	16 (2.33)	233 (8.05)	31 (17.32)	<0.001
Dog epithelium	19 (2.77)	124 (4.29)	14 (7.82)	0.009
**Ingested allergens**				
Egg	326 (43.18)	1,422 (49.48)	37 (20.56)	<0.001
Cow's milk	353 (46.75)	1,049 (36.50)	22 (12.22)	<0.001
Wheat flour	86 (11.39)	382 (13.29)	7 (3.89)	<0.001
Crab	52 (6.89)	117 (4.07)	5 (2.78)	0.002
Shrimp	36 (4.77)	63 (2.19)	1 (0.56)	<0.001
Peanut	20 (2.65)	51 (1.77)	0 (0.00)	0.048
Soybean	28 (3.71)	33 (1.15)	0 (0.00)	<0.001

Total positivity uses age-group denominators 1,030/2,950/186. Allergen-specific rates use tested n: inhalant 686/2,893/179; ingested 755/2,874/180. FDR q values are in Section 3.4.

**Table 4:** Distribution of the number of sensitizing allergens by age group [*n* (%)].

Number of sensitizations	<3 years	3 to <12 years	12 to <18 years	Total
0	425 (41.26)	525 (17.80)	56 (30.11)	1,006 (24.15)
1	263 (25.53)	484 (16.41)	24 (12.90)	771 (18.51)
2 to 4	284 (27.57)	1,330 (45.08)	81 (43.55)	1,695 (40.69)
5 to 7	49 (4.76)	546 (18.51)	24 (12.90)	619 (14.86)
≥8	9 (0.87)	65 (2.20)	1 (0.54)	75 (1.80)

The number of sensitizations was defined as the count of allergens with sIgE ≥ class 1 among the 20 allergens tested. Overall *χ*^2^ = 379.432, *P* < 0.001.

### 3.5 Multivariable logistic regression

**Figure 2:**
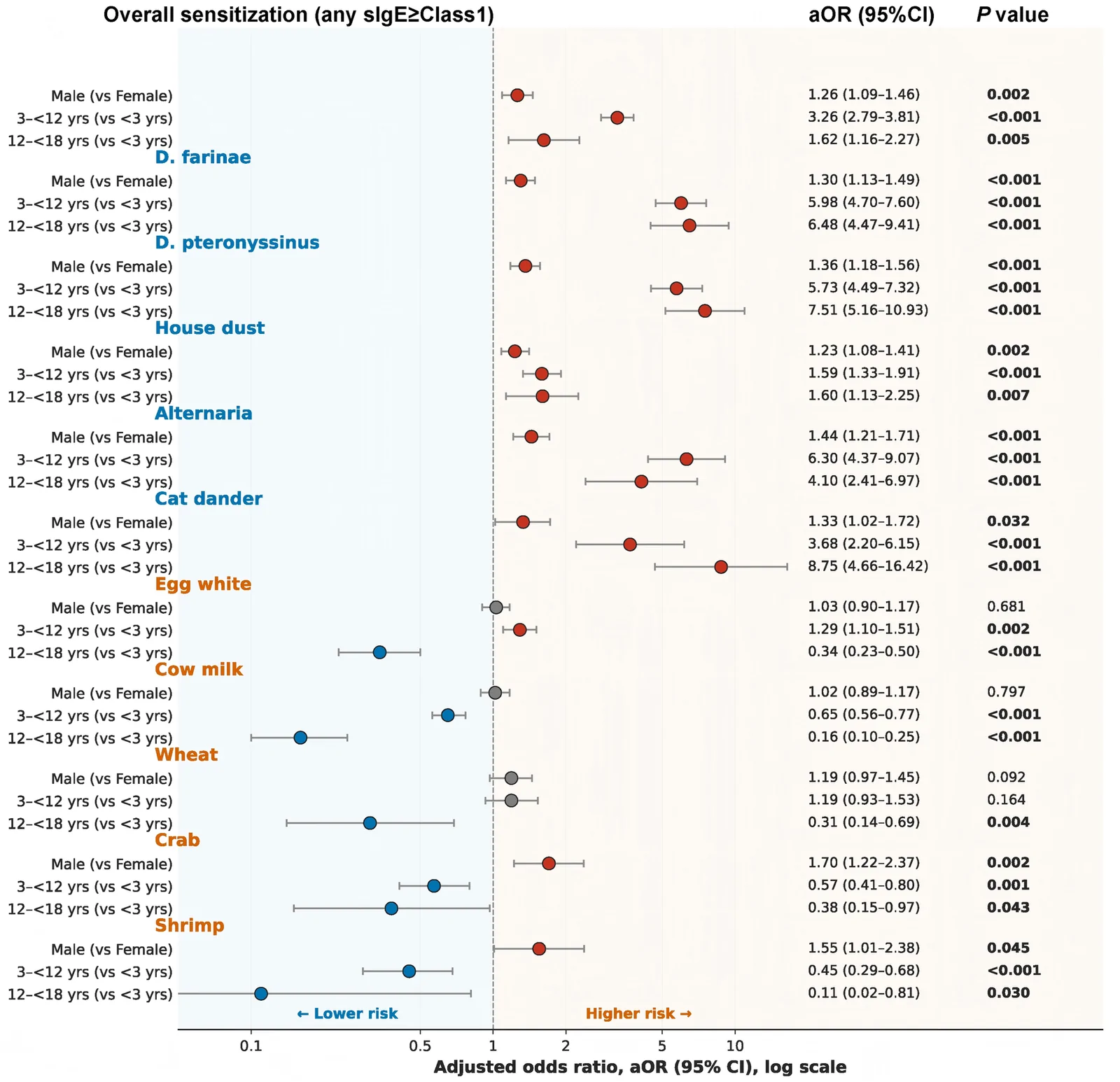
Forest plot of multivariable logistic regression for overall sensitization and for 10 major allergens. Each row presents the adjusted odds ratio (aOR, dot) with its 95% CI (horizontal bar): red indicates a significant aOR > 1, blue a significant aOR < 1, and grey a non-significant result. “Significant” denotes *P* < 0.05 for that exposure term; model *n* is 4,166 for total sensitization, 3,758 for inhalant allergens, and 3,809 for ingested allergens, with subgroup events and tested *n* in Tables 1, 3.

Using children <3 years and girls as references, male sex was an independent risk factor for overall sensitization (aOR 1.26, 95% CI 1.09 to 1.46; *P* = 0.002). The aORs for overall sensitization in the 3 to <12 years and 12 to <18 years groups were 3.26 (2.79 to 3.81) and 1.62 (1.16 to 2.27), respectively. Single-allergen analyses revealed clear age-related patterns. Sensitization to *D. pteronyssinus*, *D. farinae*, *A. alternata*, and cat epithelium increased with age. In the 12 to <18 years group the aOR for *D. pteronyssinus* rose to 7.51 (5.16 to 10.93) and that for cat epithelium to 8.75 (4.66 to 16.42). Conversely, the risk of sensitization to cow's milk, egg, and wheat flour decreased with age; in the 12 to <18 years group, the aOR for cow's milk fell to 0.16 (0.10 to 0.25) and that for egg to 0.34 (0.23 to 0.50). Male sex independently increased the risk of sensitization to *D. pteronyssinus*, *D. farinae*, house dust, cat epithelium, *A. alternata*, shrimp, and crab, with aORs ranging from 1.23 to 1.70 (Table 5**,** Figure 2). The model for total sensitization showed adequate fit (HL *χ*^2^ = 1.82, df = 4, *P* = 0.77), no collinearity (VIF ≤ 1.13), and no sex-by-age interaction (*P* = 0.40). Adding diagnostic category left sex and age estimates essentially unchanged (male aOR 1.24; 3–<12 y 2.97; 12–<18 y 1.58). *Post-hoc* power was 43% for the observed adolescent mite difference; non-significant adolescent findings are inconclusive.

**Table 5:** Multivariable logistic regression of overall and major-allergen sIgE positivity (*n* = 4,166).

Dependent variable	Exposure variable	aOR (95% CI)	*P*
**Total positivity**	Male	1.26 (1.09 to 1.46)	0.002
	3 to <12 years	3.26 (2.79 to 3.81)	<0.001
	12 to <18 years	1.62 (1.16 to 2.27)	0.005
*Dermatophagoides farinae*	Male	1.30 (1.13 to 1.49)	<0.001
	3 to <12 years	5.98 (4.70 to 7.60)	<0.001
	12 to <18 years	6.48 (4.47 to 9.41)	<0.001
*Dermatophagoides pteronyssinus*	Male	1.36 (1.18 to 1.56)	<0.001
	3 to <12 years	5.73 (4.49 to 7.32)	<0.001
	12 to <18 years	7.51 (5.16 to 10.93)	<0.001
*Alternaria alternata*	Male	1.44 (1.21 to 1.71)	<0.001
	3 to <12 years	6.30 (4.37 to 9.07)	<0.001
	12 to <18 years	4.10 (2.41 to 6.97)	<0.001
Cat epithelium	Male	1.33 (1.02 to 1.72)	0.032
	12 to <18 years	8.75 (4.66 to 16.42)	<0.001
Egg	3 to <12 years	1.29 (1.10 to 1.51)	0.002
	12 to <18 years	0.34 (0.23 to 0.50)	<0.001
Cow's milk	3 to <12 years	0.65 (0.56 to 0.77)	<0.001
	12 to <18 years	0.16 (0.10 to 0.25)	<0.001
Crab	Male	1.70 (1.22 to 2.37)	0.002
	12 to <18 years	0.38 (0.15 to 0.97)	0.043
Shrimp	Male	1.55 (1.01 to 2.38)	0.045
	12 to <18 years	0.11 (0.02 to 0.81)	0.030

Each model was adjusted for sex and age group simultaneously; female sex and age <3 years were the reference categories. Allergens with at least one significant term are shown, including non-significant terms. Model n: total 4,166; inhalant 3,758; ingested 3,809. Diagnostics in Section 3.5.

### 3.6 Co-sensitization patterns of major allergens

**Figure 3:**
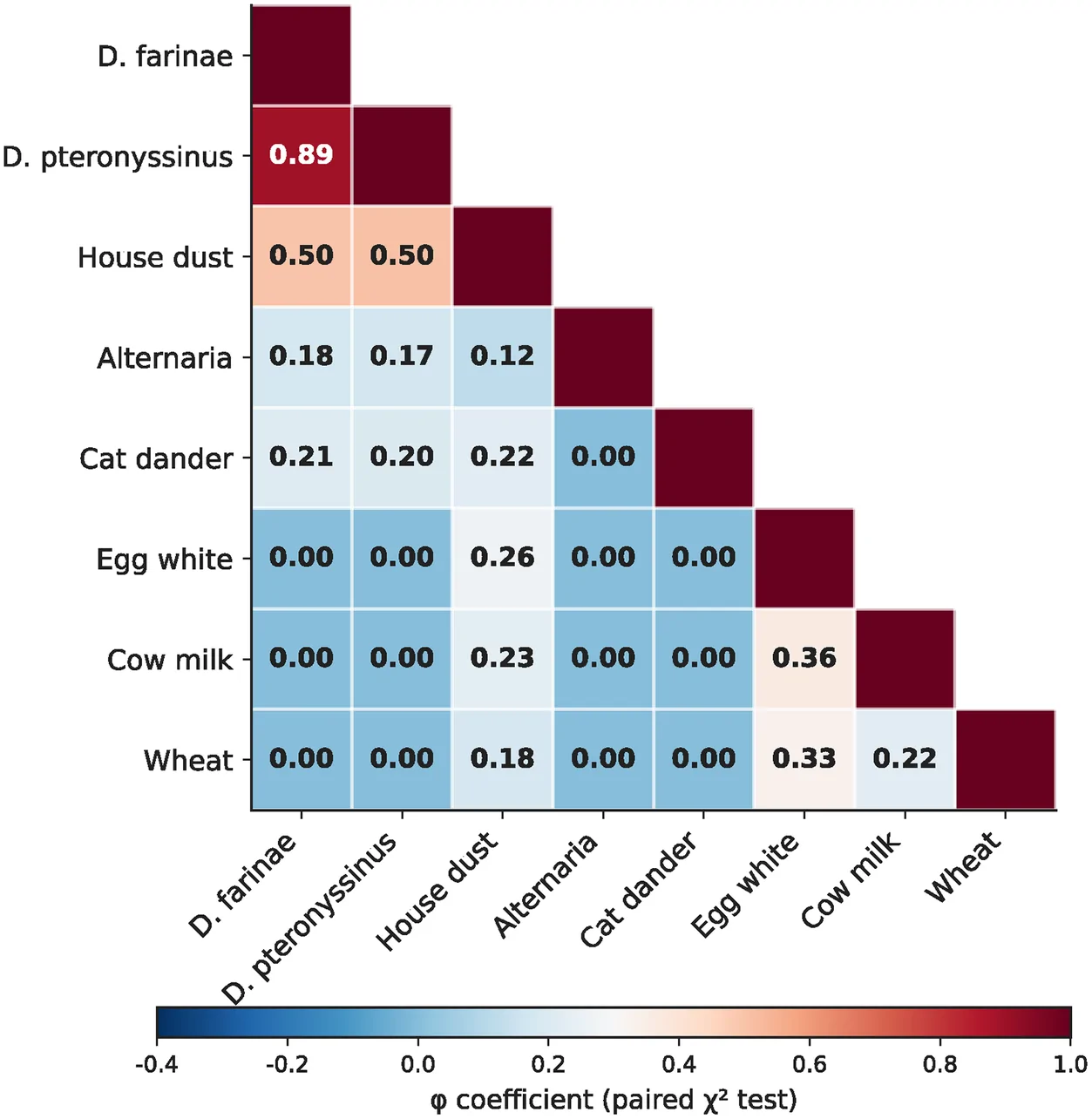
Co-sensitization structure of major allergens. Pairwise *φ*-coefficient correlation matrix (lower-triangular form) for eight major allergens; the *φ* coefficient was derived from paired *χ*^2^ tests. Three distinct clusters were identified: a mite cluster, a house-dust spillover cluster, and an early-food cluster.

*φ*-coefficient analysis identified three distinct co-sensitization clusters (Table 6**,** Figure 3). (1) **Mite cluster:** *D. farinae* and *D. pteronyssinus* showed a very strong correlation (*φ* = 0.888, with 1,338 dual-positive cases). (2) **House-dust spillover cluster:** house dust was strongly correlated with *D. pteronyssinus* (*φ* = 0.503) and with *D. farinae* (*φ* = 0.500), suggesting that house-dust sensitization is predominantly driven by mite antigens. (3) **Early-food cluster:** egg with cow's milk (*φ* = 0.361), egg with wheat flour (*φ* = 0.330), and cow's milk with wheat flour (*φ* = 0.218) showed moderate correlations. Cat epithelium–mite associations (*φ* = 0.201 to 0.219) lay at the lower boundary of the moderate range, whereas A. alternata–mite associations A. alternata(*φ* = 0.174 to 0.179) fell just below the 0.20 threshold.

**Table 6:** Co-sensitization between major allergens: *φ*-coefficient analysis.

Allergen A	Allergen B	A positive (n)	B positive (n)	Dual-positive (n)	*φ* coefficient	*P*
*Dermatophagoides farinae*	*Dermatophagoides pteronyssinus*	1,480	1,409	1,338	0.888	<0.001
*Dermatophagoides pteronyssinus*	House dust	1,409	1,433	956	0.503	<0.001
*Dermatophagoides farinae*	House dust	1,480	1,433	983	0.500	<0.001
Egg	Cow's milk	1,785	1,424	963	0.361	<0.001
Egg	Wheat flour	1,785	475	420	0.330	<0.001
House dust	Egg	1,433	1,785	872	0.263	<0.001
House dust	Cow's milk	1,433	1,424	706	0.230	<0.001
House dust	Cat epithelium	1,433	280	205	0.219	<0.001
Cow's milk	Wheat flour	1,424	475	299	0.218	<0.001
*Dermatophagoides farinae*	Cat epithelium	1,480	280	204	0.209	<0.001
*Dermatophagoides pteronyssinus*	Cat epithelium	1,409	280	194	0.201	<0.001
*Dermatophagoides farinae*	*Alternaria alternata*	1,480	739	399	0.179	<0.001
*Dermatophagoides pteronyssinus*	*Alternaria alternata*	1,409	739	381	0.174	<0.001

The φ coefficient is interpreted following Cohen's conventional thresholds: |φ| ≥ 0.20 indicates a moderate correlation; |φ| ≥ 0.50 indicates a strong correlation. Cat–mite associations (φ = 0.201–0.219) lie at the lower boundary of the moderate range; Alternaria–mite associations (φ = 0.174–0.179) fall just below this threshold.

## 4 Discussion

In this single-center retrospective cross-sectional study of 4,166 children with allergic diseases, we characterized sIgE sensitization to 20 common allergens. Sensitization was dominated by HDM and early food allergens, with male predominance for inhalant and crustacean sensitization, an age-related shift from food to inhalant allergens, and distinct co-sensitization patterns. The following sections compare these findings with regional data and discuss their mechanisms, clinical implications, and limitations.

### 4.1 Major allergen composition and comparison with other Chinese populations

The overall sensitization rate of 75.85% and the top-five composition of egg, *D. farinae*, house dust, *D. pteronyssinus*, and cow's milk observed in our cohort align closely with previous studies in eastern China. A study of 39,926 children at the Shanghai Children's Medical Center (11) and a study of children with allergic rhinitis in Changzhou (12) both showed that mites and early food allergens dominate the sensitization spectrum. By contrast, a study of children aged 0 to 14 years in the Kashgar prefecture of Xinjiang reported an overall sensitization rate of only 42.7%, with tree pollens and cat dander dominating the top five (13), underscoring how strongly the local sensitization spectrum is shaped by climate and vegetation. HDM are the dominant perennial indoor allergens in humid regions (14). Recent district-level data from Suzhou also showed HDM-dominant and age-dependent sensitization patterns in a nearby secondary-care population (15). The strong co-sensitization between *D. farinae* and *D. pteronyssinus* (*φ* = 0.888) likely reflects the high structural homology and >80% IgE cross-reactivity between their major allergens, including Der p 1/Der f 1 and Der p 2/Der f 2 (16, 17). House-dust sensitization was predominantly low-grade and strongly correlated with both mite species (*φ* ≈ 0.50), suggesting that it largely reflects mite-derived antigens. Because whole-dust extracts contain multiple antigens at relatively low concentrations, house-dust sIgE may remain low even in mite-sensitized children and should therefore be interpreted as a low-resolution surrogate for mite sensitization (17). *A. alternata* sensitization was observed in 19.66% of children and has been associated with moderate-to-severe persistent asthma (18), supporting closer clinical attention in sensitized patients.

### 4.2 Ingested allergens and the particular case of shellfish

Egg and cow's milk showed high but predominantly low-class sIgE positivity, with positivity declining significantly with age, consistent with the natural development of tolerance to these allergens (19, 20). Importantly, sIgE positivity does not necessarily indicate clinical allergy; sensitization to egg or cow' s milk is considerably more common than clinically confirmed allergy in children with atopic dermatitis or asthma (7, 21). Therefore, unnecessary dietary restriction should be avoided in children <3 years with low-class food sensitization to minimize potential adverse effects on growth and development (19). Although crustacean sensitization was uncommon (crab 4.57%; shrimp 2.63%), high-class sensitization was relatively frequent among positive cases (12.1% and 13.0%, respectively), and male sex was independently associated with both crab and shrimp sensitization (aOR 1.70 and 1.55, respectively). Tropomyosin, a major crustacean allergen, shares substantial sequence homology and IgE-binding epitopes with the mite allergen Der p 10 (22, 23), suggesting that some crustacean sensitization may reflect mite–shellfish cross-reactivity. Positive crustacean sIgE should therefore be interpreted alongside dietary exposure and clinical history, particularly when high-class sensitization is accompanied by a clear reaction history (23).

### 4.3 Sex, age-related spectrum migration, and polysensitization

Boys showed higher sensitization to several inhalant and crustacean allergens, whereas no sex difference was observed for major food allergens. This pattern is consistent with the BAMSE birth cohort, in which male sex was associated with airborne but not food allergen sensitization (24). Sex-related differences in Th2 responses and mast-cell activation may partly contribute to the male predominance in inhalant sensitization during childhood and adolescence (25).

The shift from food-dominant sensitization in children <3 years to inhalant-dominant sensitization in adolescents was consistent with the atopic-march framework (6, 7). Early food sensitization has been associated with subsequent asthma and allergic rhinitis (7, 26), supporting follow-up for emerging respiratory atopy in young sensitized children (27–29). Because this study was cross-sectional, individual sensitization trajectories could not be assessed. The lower overall sensitization rate in children <3 years likely reflected both incomplete panel coverage and shorter cumulative aeroallergen exposure. Co-sensitization based on whole extracts may reflect both true polysensitization and cross-reactivity; mite–house-dust and crustacean–mite associations are plausibly driven by shared antigens, whereas mite–*A. alternata* associations may reflect shared environmental exposure and atopic predisposition. Given the high prevalence of polysensitization, children with multiple inhalant sensitizations may warrant closer attention to asthma control.

### 4.4 Clinical and public-health implications, and limitations

Building on the findings above, this study has several implications for pediatric allergic-disease care at county-level cities. Sensitization profiles showed some consistency with clinical diagnoses, with higher mite and inhalant sensitization in children with respiratory diagnoses, while food sensitization remained common in both groups. However, sIgE positivity alone does not establish clinical allergy. First, *D. pteronyssinus* and *D. farinae* should serve as the core inhalant allergens for screening; given their very high co-sensitization, either species can be used as a primary screening marker when resources are limited, but both should be tested when AIT is being considered (30). Second, children <3 years with low-class food sensitization should not be subjected to overly restrictive diets; clinical decisions should integrate feeding history and symptom follow-up, with oral food challenge used to confirm clinical allergy when needed (19, 31). Third, for children with moderate-to-severe or poorly controlled mite-driven allergic rhinitis or asthma, the EAACI guidelines recommend HDM-AIT as add-on therapy (30); the 49.72% *D. pteronyssinus* positivity in our 12 to <18 years subgroup suggests a substantial pool of potential AIT candidates in this region. Fourth, environmental interventions, including regular laundering of bedding, remain a reasonable component of primary prevention (18, 30). Given the non-interchangeability of EIA and ImmunoCAP grades, absolute grade thresholds derived from ImmunoCAP-based protocols should not be directly applied to the HB-500E platform. Because house dust is a composite extract and showed predominantly lower-grade sensitization in our cohort, house-dust sIgE should not be used alone to guide AIT decisions and should instead be interpreted alongside mite-specific sensitization and the clinical phenotype.

Several limitations should be taken into account when interpreting these findings. First, as a single-center, hospital-based, retrospective cross-sectional study, the cohort consisted entirely of children already presenting with allergic disease, introducing selection bias; the cohort was also diagnostically heterogeneous because diagnoses were based on primary encounter codes, although the diagnosis-stratified and sensitivity analyses did not suggest material bias, the 75.85% sensitization rate should not be extrapolated to the general community pediatric population. Second, the absence of a non-allergic comparator group precludes direct assessment of the predictive value of sIgE positivity for clinical allergy diagnosis; the principle that sIgE positivity is not equivalent to clinical allergy must therefore be emphasized when interpreting the results. Third, the 3 to <12 years group spanned a relatively broad age range, which may have obscured finer within-group age-related trends, while the 12 to <18 years group was relatively small (*n* = 186), limiting the statistical resolution for adolescence and for any reversal phenomena of spectrum migration. The study also did not capture key exposure variables such as family history of atopy, breastfeeding, timing of complementary feeding, pet ownership, and indoor dampness, precluding deeper multifactorial attribution. Fourth, sIgE was measured at the whole-allergen level for 20 allergens and did not include component-resolved diagnostics (CRD), limiting the ability to distinguish genuine sensitizing proteins from cross-reactive proteins. Fifth, systematic differences exist between the EIA platform used in this study and the internationally widespread ImmunoCAP platform; because no local head-to-head comparison was available, direct numerical comparison with ImmunoCAP-based studies is precluded and all such comparisons should be read as qualitative (32). In addition, retaining only the first sIgE record may bias severity selection, therapy-related exclusions may make high-class and polysensitization rates conservative, and the large number of parallel comparisons means that borderline *P* values should be interpreted cautiously.

## 5 Conclusion

In this county-level cohort of 4,166 children from the northern Yangtze River Delta, sIgE sensitization was dominated by house dust mites and early food allergens, with an age-related shift from food to inhalant allergens, male predominance in inhalant sensitization, and three distinct co-sensitization clusters. These findings may inform allergen screening and pediatric allergy management in similar regional settings. Multicenter prospective studies incorporating oral food challenges and component-resolved diagnostics are needed to validate these findings.

## Data Availability

The original contributions presented in the study are included in the article/Supplementary Material, further inquiries can be directed to the corresponding author.
